# Systemic lupus erythematous presenting with hemorrhagic shock caused by gastric penetration of pancreatic pseudocyst: a case report

**DOI:** 10.1186/s13256-021-03074-z

**Published:** 2021-09-18

**Authors:** Hideya Itagaki, Suzuki Katuhiko

**Affiliations:** Department of General Surgery, Honjoudaiichi Hospital, 110, Iwabuchishita, Yurihonnjou, Akita 015-8567 Japan

**Keywords:** Acute pancreatitis, Systemic lupus erythematous, Pancreatic cyst, Stomach penetration, Hemorrhagic shock

## Abstract

**Background:**

Systemic lupus erythematous that causes various organ damage is rarely associated with pancreatic lesion. To the best of our knowledge, no cases presenting with hemorrhage shock caused by gastric penetration of pancreatic pseudocyst due to lupus pancreatitis have been reported. Herein, we report a case of hemorrhage shock caused by gastric penetration of pancreatic pseudocyst due to lupus pancreatitis.

**Case presentation:**

A 53-year-old Japanese man with a history of systemic lupus erythematous, pancreatic pseudocyst, and chronic pancreatitis complained of epigastric pain and had hematemesis. He visited our emergency room and was admitted in our hospital. Upper endoscopy showed that hemostasis was obtained; however, computed tomography scan was performed since he was suspected to have gastric penetration into hollow viscera. The computed tomography revealed accumulation of fluid around the pancreas and gastric penetration of pancreatic cyst. Blood test showed increased serum amylase level. These results suggest that the exacerbation of chronic pancreatitis causes the penetration. Surgery was considered; however, we took a wait-and-see approach since hemostasis was obtained. After that, he was in stable condition, although he suffered from fever and accumulation of left pleural effusion was observed by computed tomography. However, he had massive hematemesis and melena 9 days after hospitalization and died in spite of several treatments including blood transfusion. Autopsy revealed that he actually had pleural thickening, which is not caused by accumulation of left pleural effusion but by severe pleural inflammation. We therefore performed additional blood and urinary tests on the same day. The test results showed that he had a high titer of anti-double-stranded deoxyribonucleic acid (DNA) antibody, hypocomplementemia, and erythrocyturia, indicating that he had systemic lupus erythematous with high disease activity considering his fever and pleural inflammation.

**Conclusions:**

Patients who have systemic lupus erythematous with high disease activity have the potential to develop fatal complications due to pancreatitis, so appropriate treatments are required for such patients.

## Background

Systemic lupus erythematous (SLE) is an autoimmune disease. In patients with SLE, a complex consisting of deoxyribonucleic acid (DNA) and anti-DNA antibody is formed in circulation and is deposited in tissues, causing various organ damage. Fifty percent of SLE patients have digestive symptoms [2] and rarely develop pancreatitis. The incidence of SLE-associated pancreatitis ranges from 0.9% to more than 5% of patients [1]. The pathogenic mechanism underlying pancreatitis in SLE patients is unclear. It is suggested that vasculitis and thrombosis cause pancreatitis [3]. In addition, 38% of SLE patients with pancreatitis develop chronic pancreatitis in 2 years after two episodes of acute pancreatitis [4]. Pancreatic pseudocyst is formed in 10–30% of SLE patients with acute and chronic pancreatitis [5–7]. In this study, the patient had chronic pancreatitis and pseudocyst after the development of acute pancreatitis. The episodes of acute pancreatitis resulted in gastric penetration of the pseudocyst, causing death due to hemorrhagic shock. The results of blood and urinary tests after the death showed that he had SLE with high disease activity, which caused pancreatitis. Sufficient caution and appropriate treatments are necessary for pancreatitis in SLE patients with high disease activity as in this case.

## Case presentation

**Fig. 1:**
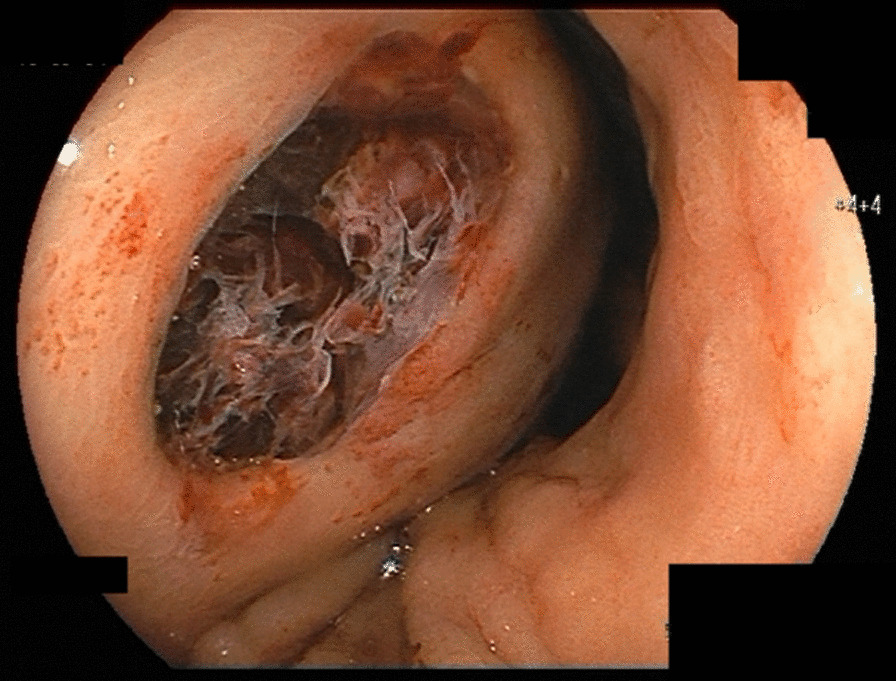
Ulcer on the submucosal tumor-like lesion. An ulcer had formed on the submucosal tumor-like lesion and was bleeding from it

**Fig. 2:**
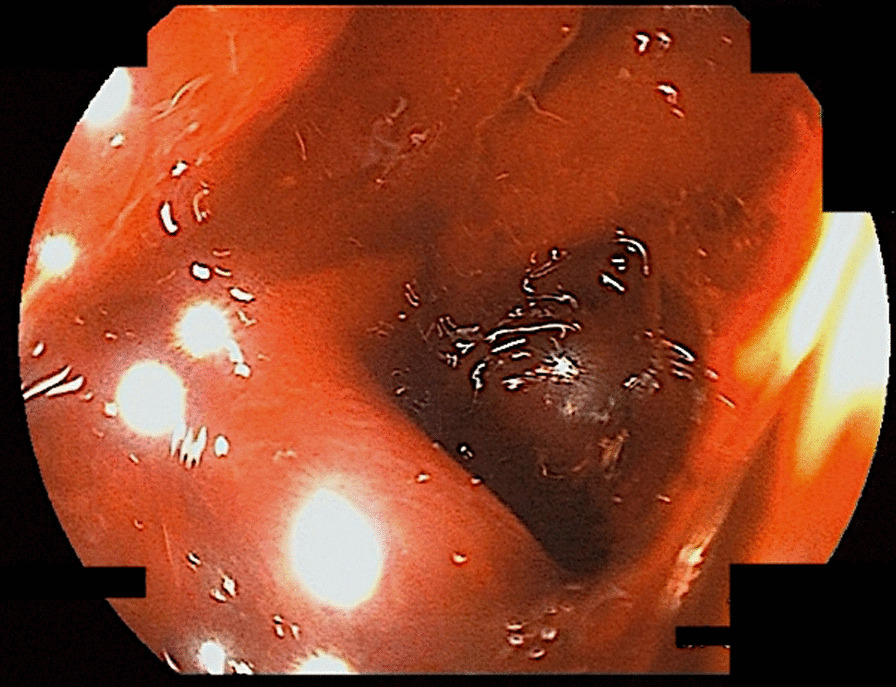
Penetration of pancreatic cyst into the stomach. The endoscope was inserted into the perforated area, and it was perforated by a pancreatic cyst

**Fig. 3:**
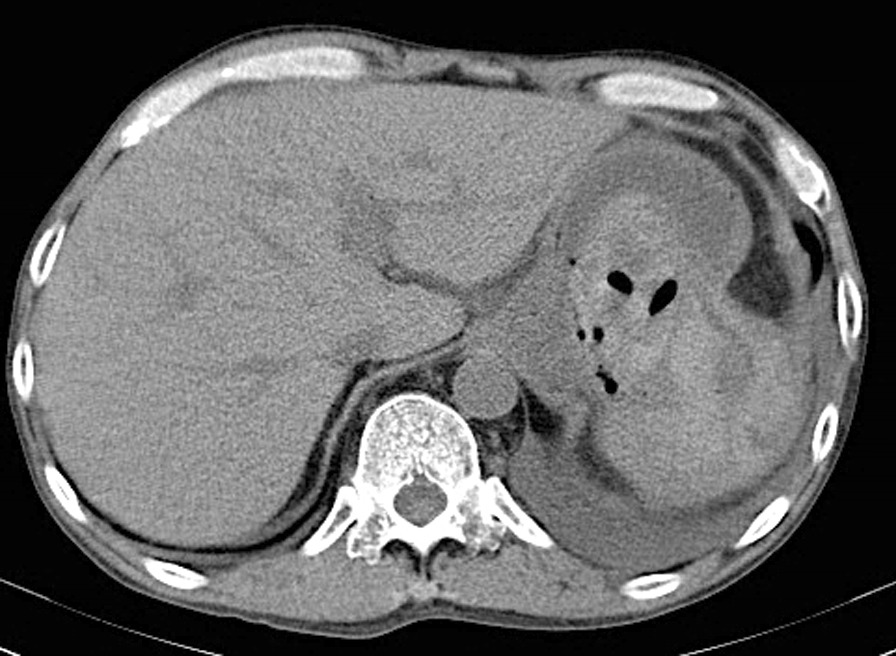
Penetration of pancreatic cyst into the stomach on CT. On CT, a clot in the stomach and a fistula to a pancreatic cyst were identified

**Fig. 4:**
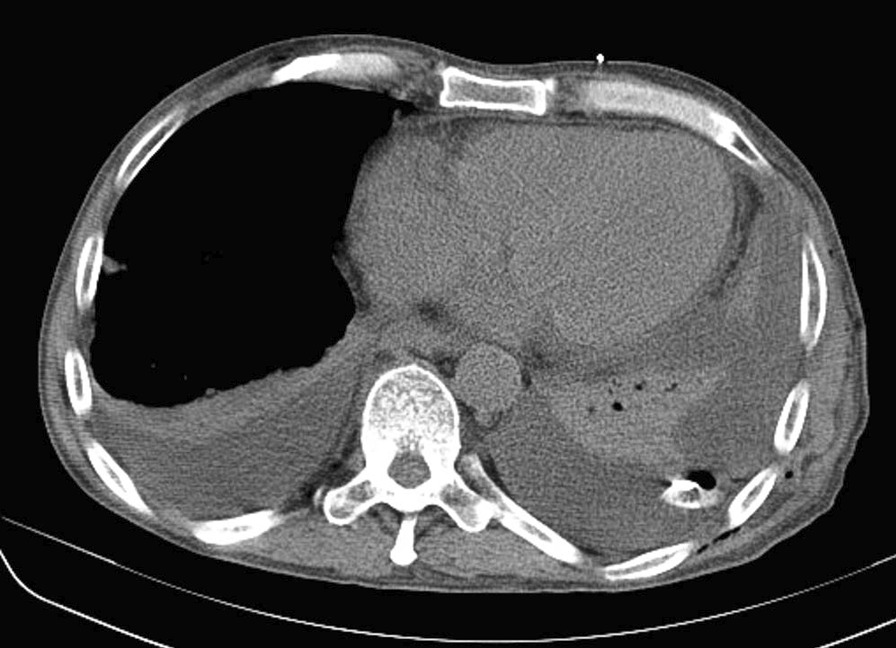
CT iamge of the thoracic drain insertion. A thoracic drain was placed in the chest cavity, but the pleural effusion was not drained.

A 53-year-old Japanese man who complained of epigastric pain and hematemesis visited our emergency room. The patient had no particular family history, but a history of SLE, pancreatic pseudocyst, and chronic pancreatitis. He took 5 mg of prednisolone, camostat mesilate, and berizym. He had epigastric pain since midnight of the day of visit, and vomited blood five to six times an hour after the onset. Upper endoscopy performed after the visit revealed that an ulcer was formed on a submucosal tumor-like lesion (Fig. 1). He bled from the ulcer, and the bleeding was stopped using thrombin. Upper endoscopy on the next day showed that the bleeding from the ulcer was stopped. However, he was suspected to have penetration into a hollow viscus after the removal of clots (Fig. 2). We performed computed tomography (CT) scan analysis to identify the hollow viscus. The CT result revealed the accumulation of fluid around the pancreas and penetration of pancreatic cyst into the stomach (Fig. 3). Blood test performed on the same day indicated that the serum amylase level was 164 U/L, which is higher than normal level. Considering the CT image finding, the patient was diagnosed as gastric penetration caused by acute pancreatitis episode in chronic pancreatitis. We considered surgery, but we decided that we can take a wait-and-see approach, since hemostasis was confirmed on the upper endoscopy. He had abdominal pain 5 days after hospitalization, and blood test and CT scan were performed. The blood test result showed a slight increase in inflammation and no elevation of serum amylase level. The CT scan result indicated no new findings but revealed left pleural effusion. We suspected that he might have secondary pleural effusion. However, since we cannot deny the possibility that he had pancreatic cyst and diaphragmatic penetration, the thoracostomy tube was inserted 7 days after hospitalization. Only a small amount of pleural effusion was obtained, and the amylase level in the pleural effusion was normal. To identify the cause for the small amount of pleural effusion, we performed CT scan analysis 8 days after hospitalization. The CT result revealed that there was no problem with the insertion position of the thoracostomy tube, and the cause for the small amount of pleural effusion remained unclear (Fig. 4). We took a wait-and-see approach, since he had fever but had been stable up to this point. However, he had massive hematemesis and melena 9 days after hospitalization, and his condition rapidly deteriorated. Blood transfusion was performed but had no effect, and he died on that day. Autopsy performed on the day of death showed that the large amount of bleeding was not from an aneurysm but from the cyst wall, and revealed that he actually had pleural thickening, which is not caused by accumulation of left pleural effusion but by severe pleural inflammation, indicating that he had pleural inflammation. In addition, accumulation of cardiac effusion was also observed, and he was suspected to have pericardium inflammation. We therefore performed additional blood and urinary tests 9 days after hospitalization. The test results were as follows: platelet count, 8.1 10^4^/UL; anti-dsDNA antibody titer, 92.8 IU; complement component, C3, 29 mg/dL; C4, 4 mg/dL; CH50 < 10 U/mL; red blood cell count in urine, 20–29 per high-power field (HPF). Considering that he had fever, pleural inflammation, and pericardium inflammation, it turned out that SLE had high disease activity in this patient, resulting in bleeding.

## Discussion

Our intervention for this patient was delayed, because two rare events occurred. One is that gastric penetration of pancreatic pseudocyst caused hemorrhagic shock. The other one is that the cause of the gastric penetration was lupus pancreatitis (LP) due to SLE.

We first discuss the LP and hemorrhagic shock. Only 1.3% of LP patients develop hemorrhagic shock, which is very rare. The causes of hemorrhagic shock include the pancreatic cyst wall, pseudoaneurysm of the splenic artery, and rupture of splenic artery in some cases [8, 9]. Hemorrhagic shock caused by bleeding from the cyst wall is found to be obvious by autopsy in this case, and it is often said that bleeding from the cyst wall occurs in 6–17% of patients with chronic pancreatitis, with case fatality rate being 40% without early intervention. Pseudopancreatic cysts and the gastrointestinal tract can also form fistulas and cause upper or lower gastrointestinal bleeding [10, 11]. Araki *et al.* reported that the penetration of the pancreatic cyst wall into the gastrointestinal tract and gut tract causes hematemesis, as in this patient [12]. They also reported that the basic treatments for bleeding due to pancreatitis are transcatheter arterial embolization (TAE) and surgery such as gastrectomy, pancreatectomy, and splenectomy and that they performed TAE first and then surgery for a patient with pseudoaneurysm and rupture of artery [12]. Regarding treatment options, TAE and surgery are assumed as described above, but it is often said that the mortality rate is 20–29% when surgery is performed in hemodynamically unstable patients with cyst rupture included in this study. Thus, TAE, but not surgery, is considered to be first-line treatment [10]. Actually, we considered TAE and surgery for the patient in this study, but we had difficulty determining the right timing of surgery and TAE. The reason for this is that hemostasis was already obtained at the time of hospitalization and that the patient’s condition rapidly deteriorated after rebleeding. This patient might not be treatable with TAE, since no development of obvious aneurysm was observed at autopsy and the bleeding seemed to be from the cyst wall. In addition, a study reported that no rebleeding or patient death occurred after the surgery, thus recommending its choice [13]. It might have been preferable that we perform the surgery, that is, pancreatectomy, for the patient in this study during hemostasis condition.

We next discuss SLE and pancreatitis in this patient. SLE causes various organ damage, and pancreatitis is one of the fatal complications. The average age of onset of pancreatitis caused by SLE is 30 years, and female patients are significantly more common than male patients (90% of patients are female) [14, 15]. Its prevalence rate is estimated to 0.9–4% [15, 16]. Forty-four percent of patients develop pancreatitis caused by SLE (lupus pancreatitis, LP) within a year of diagnosis of SLE [14]. The causes of LP are suggested to be vasculitis and embolism, and the most common cause is mesenteric vasculitis [17]. The risk factors of LP include gallstones, alcohol consumption, infection, and hypertriglyceridemia as well as common pancreatitis [15]. In particular, 76.9% of LP patients have hypertriglyceridemia [18]. The disease activity of SLE is high in many SLE patients with pancreatitis, and it is correlated with the high SLEDAI score [15, 19, 20]. A study reported that the average SLEDAI score of LP patients was 21.70, which suggests that its disease activity is high [19]. In addition, various organ damage occurs in many LP patients, and patients frequently have nephritis, pleural effusion, and arthritis as symptoms [3]. The mortality of LP patients is more than 20–30%, which is very high [14, 19]. Concomitant infection and thrombocytopenia are its poor prognosis factors [18]. LP and common pancreatitis are usually treated with supportive care. To treat SLE patients with high disease activity, supportive care is coupled with using high-dose steroid, cyclophosphamide, and azathioprine [15, 18]. The mortality of LP patients treated with steroid was significantly lower than that of those in the non-steroid treatment group (20% and 61%, respectively) [14]. However, azathioprine use requires particular attention, since a study reported that azathioprine caused pancreatitis in some cases [8]. The mortality of patients treated with steroid and azathioprine was lower than that of patients not treated with these drugs [14]. These studies suggest that it is necessary to select patients who benefit from treatment with azathioprine.

This patient is not a typical SLE patient based on age and gender. However, he had nephritis, pleuritic, pericarditis, hypocomplementemia, high titer of anti-dsDNA antibody, and thrombocytopenia. His SLEDAI score was 14 points, indicating that he had SLE with high disease activity. There are two reasons for the death of this patient. One is that we did not think that he had LP. The other one is that we believed we could control disease progression during the events, since we successfully controlled SLE disease progression in this patient with low dose of prednisolone (5 mg). We should have used high-dose steroid for this patient.

## Conclusion

To the best of our knowledge, no cases presenting with hemorrhage shock caused by gastric penetration of pancreatic pseudocyst due to LP have been reported. We need to consider the possibility that SLE patients with pancreatitis have LP. Earlier treatment using steroid is required. In addition, SLE patients with pancreatitis rarely have bleeding. The basic treatments for bleeding are TAE and surgery in such patients. However, we need to consider surgery for the patients with rebleeding.

## Data Availability

Data sharing is not applicable to this article as no datasets were generated or analyzed during the current study.

## Abbreviations

SLE: Systemic lupus erythematous
LP: Lupus pancreatitis
TAE: Transcatheter arterial embolization
